# Letter to the Editor in Response to ‘The Effect of Polycystic Ovarian Syndrome on Development of Diabetes Mellitus by a Multistate Modelling Approach’

**DOI:** 10.1002/edm2.70346

**Published:** 2026-09-15

**Authors:** Saad Nawaz

**Affiliations:** ^1^ Liaquat University of Medical and Health Sciences Jamshoro Pakistan

To the Editor,

1

We read with interest the study by Mousavi et al. [1] applying a multistate Markov model to examine how polycystic ovary syndrome (PCOS) influences the progression from normoglycemia to pre‐diabetes and diabetes. Using a multistate framework to track changes in blood sugar levels is a real improvement over earlier studies that looked at only one point in time, and we commend the authors for this contribution. We would like to raise three points that deserve further consideration.

First, the final analytic sample represents only about a quarter of the women who were originally free of diabetes and pre‐diabetes at baseline. Of 6493 eligible women, more than 4900 were excluded—most commonly for undocumented PCOS status or for having only isolated PCOS features that did not meet the full Rotterdam criteria. Exclusion on this scale can change who a study is actually describing, since the women who remain may differ systematically from those who were excluded [2]. We think the paper would benefit from a comparison of baseline characteristics between included and excluded women, so readers can judge how much this narrowing affects the generalizability of the reported hazard ratios.

Second, we are concerned that the study's central finding—a 23% higher hazard of transitioning from normoglycemia to pre‐diabetes among women with PCOS—may partly reflect detection rather than true biological risk. The authors themselves note that women with PCOS typically receive closer clinical follow‐up. Because pre‐diabetes is an actively screened‐for state, more frequent testing in this group could produce more diagnoses even without any real difference in underlying risk, a phenomenon well documented as surveillance bias in other chronic disease settings [3]. This is especially important here because PCOS did not significantly predict the other two transitions, a pattern that is at least as consistent with differential detection as with a genuine PCOS‐specific effect confined to the earliest disease stage.

Third, the strength of the primary result itself deserves a closer look. The reported hazard ratio of 1.23 has a 95% confidence interval of 1.01–1.49, sitting only just above the null, and it emerges from a table of roughly two dozen hazard ratio estimates tested without any stated correction for multiple comparisons. Confidence intervals that barely reach 1.0 are by definition weak, and when several relationships are analysed at a traditional significance level, some will look significant by chance alone [4]. We would welcome the authors' comments on whether the finding holds up under a stricter significance threshold or a sensitivity analysis adjusting for the number of comparisons made.

We raise these points not to diminish the value of the authors' work, but because we believe addressing them would strengthen confidence in the study's central claim and better guide how clinicians should interpret and act on it.

## Author Contributions

**Saad Nawaz:** conceptualization, investigation, funding acquisition, writing – original draft, writing – review and editing, visualization, validation, formal analysis, project administration.

## Funding

The author has nothing to report.

## Ethics Statement

The author has nothing to report.

## Consent

The author has nothing to report.

## Conflicts of Interest

The author declares no conflicts of interest.

## Data Availability

Data sharing not applicable to this article as no datasets were generated or analysed during the current study.
